# A lived experience response to the proposed diagnosis of terminal anorexia nervosa: learning from iatrogenic harm, ambivalence and enduring hope

**DOI:** 10.1186/s40337-022-00729-0

**Published:** 2023-01-05

**Authors:** Rosiel Elwyn

**Affiliations:** Thompson Institute, University of the Sunshine Coast, Birtinya, QLD Australia

**Keywords:** Severe-enduring anorexia nervosa, Terminal anorexia nervosa, Ethics, Euthanasia, Futility, Medical assistance in dying, Ambivalence, Lived experience

## Abstract

The ethical approach to treatment non-response and treatment refusal in severe-enduring anorexia nervosa (SE-AN) is the source of significant ethical debate, particularly given the risk of death by suicide or medical complications. A recent article proposed criteria to define when anorexia nervosa (AN) can be diagnosed as ‘terminal’ in order to facilitate euthanasia or physician-assisted suicide (EAS), otherwise known as medical assistance in dying, for individuals who wish to be relieved of suffering and accept treatment as ‘futile’. This author utilises their personal lived experience to reflect on the issues raised, including: treatment refusal, iatrogenic harm, suicidality and desire to end suffering, impact of diagnosis/prognosis, schemas, alexithymia, countertransference, ambivalence, and holding on to hope. Within debates as critical as the bioethics of involuntary treatment, end-of-life and EAS in eating disorders, it is crucial that the literature includes multiple cases and perspectives of individuals with SE-AN that represent a wide range of experiences and explores the complexity of enduring AN illness, complex beliefs, communication patterns and relational dynamics that occur in SE-AN.

## Proposed diagnosis of terminal anorexia nervosa

Preliminary criteria for ‘terminal’ anorexia nervosa (AN) was recently proposed by Gaudiani et al. [1], with three cases used to illustrate the defining criteria. It was argued that a case should be made for defining ‘terminal’ AN on the basis of identifying patients with severe-enduring anorexia nervosa (SE-AN) who may be eligible for access to palliative care and hospice care, and to facilitate access to euthanasia and physician-assisted suicide (EAS), [also known as medical assistance in dying (MAID)]. The proposed characteristics for ‘terminal’ AN included: (1) AN diagnosis (2) Age over 30, (3) Prior persistent engagement in high-quality, multidisciplinary eating disorders care, and (4) decision-making capacity to understand further treatment is futile, the wish not to prolong life and acceptance of death [1]. In response to concerns and criticism, the authors have refined their discussion and argument for the proposed diagnosis [2]. This article is written in dialogue with these two papers [1, 2].

This response is written from my perspective as an individual with living experience of SE-AN. My personal lived experience includes inpatient and outpatient treatment, and iatrogenic harm that impacted my engagement in treatment and compounded my illness course and suicidality. Identity and experiential intersections have also been important aspects of my illness prognosis and treatment engagement (neurodivergence, LGBTIQ+ identity, trauma, co-occurring psychosocial/psychiatric disability, chronic medical issues as secondary to SE-AN). I experienced significant gaps in my treatment with respect to these identity and experiential intersections, and as a consequence, my treatment was often ineffective or harmful. My lived experience can be used to reflect on issues with the concept of treatment futility in AN and potential for the proposed ‘terminal’ AN diagnosis for both care and harm.

## Lived experience: severe-enduring anorexia nervosa

My ED began at the age of 8, characterised by food and water restriction. I was repetitively self-harming (self-cutting, burning) and first attempted suicide at the age of 12. Other ED-related behaviours that emerged during these early years included giving food away and hiding food, self-denial of comfort (forced self-exposure to cold, sleeping on the floor, disengagement from enjoyable past-times, self-isolation), and wearing items that restricted movement or caused physical harm (e.g., belts or chains for self-mortification). I experienced multiple traumatic experiences throughout early childhood, adolescence and adulthood. Part of the abuse and neglect I experienced included medical neglect; I would not be taken to medical facilities after experiencing illness or injury such as broken bones, suspected concussions, chest pain, infections, allergic reactions and other health problems. These experiences shaped my ED development, course and experience of treatment—I understood medical care as something that was not accessible even in life-threatening circumstances, and I internalised this as a belief that I was unworthy of medical care when facing harm or death. Once ill with AN and other co-occurring conditions, this belief would be reinforced in harmful treatment experiences.

At the age of 9, it was identified that I was giving my food away to other children at school, dehydrating myself, and exercising compulsively. For a brief time, my meals were supervised by teachers, however no further action was taken. After a second suicide attempt at 14, I was hospitalised, and after more severe weight loss, I was referred to a Child and Youth Mental Health Services (CYMHS). Despite disclosing my ED history, I was not diagnosed nor treated for an ED: the treating team focused on my suicidality, self-harm, elective mutism and emerging psychotic experiences. At the age of 17, my ED and other mental health concerns became progressed, and I was repeatedly hospitalised for the next year for the other mental health concerns. I aged out of CYMHS, and at the age of 18, continued to deteriorate, and was hospitalised after a period of acute weight loss and the early stages of organ failure. In this hospitalisation I was first diagnosed with AN, restrictive subtype (body mass index; BMI 11). My weight was the primary focus of the admission, and I believe having a low body weight was the sole reason my ED had finally been diagnosed. Throughout my early adulthood, I was repeatedly involuntarily and voluntarily hospitalised and tried multiple forms of outpatient psychotherapeutic and psychopharmaceutical treatments, including combinations of antipsychotics, mood stabilisers, and antidepressants. I did not respond to cognitive behavioural therapy (CBT), acceptance and commitment therapy (ACT), interpersonal therapy (IPT), dialectical behaviour therapy (DBT), metacognitive therapy (MCT), art therapy, group therapy, or inpatient and outpatient hospital treatments, nor improved with medications. I did not receive a full course of family therapy as family members refused to engage with this modality.

After 16 years of illness with repeated relapse and loss of weight following inpatient nutritional restoration and weight restoration, my AN was re-diagnosed as SE-AN (also labelled by treating teams as ‘intractable’, ‘recalcitrant’, ‘non-responsive’). I have been turned away from multiple mental health and medical clinics and private practitioners on the basis of my treatment non-response and poor prognosis. Their refusal has also been based in part on the complexity of my co-occurring mental health concerns and neurodivergence: trauma history, suicide attempts, psychosis, and autism. At the time of writing, I am 31 years of age (illness duration 23 years), have a BMI of 13, and depend on nutritional supplement formulas as my primary source of nutrition. My experiences of ED treatments, and my own and clinical judgements of the (un)likeliness of my recovery have been important factors in my treatment engagement and refusal.

## Impact of diagnosis and prognosis

The authors’ briefly addressed concern that the diagnostic terminology could be demoralising, however they dismissed this criticism as “doomsdaying” that “without supporting evidence, likely doesn’t have a place in academic discussion” [2]. This is invalidation of an important aspect of lived experience realities that *do* merit a place within discussion. It is important to hold space for how a ‘terminal’ diagnosis may both be felt as empowering, validating and compassionate to some, and for others and their families, may create a sense of hopelessness. Diagnostic labelling and language can frame and shape our sense of reality, self and identity, our embodied experiences, and to direct our behaviour [3, 4]. AN may be used as a form of embodied communication of unheard and unrecognised suffering, and diagnostic labelling has an important role in this relationship. Psychiatric diagnoses may be experienced as attributing authoritative legitimacy to suffering, particularly for individuals who have been invalidated and silenced [5, 6]. AN diagnoses and scripts may inform how we relate to and ‘live up to’ expectations, symptoms, and engage with treatment [4]. By creating expectations and symbolic interactions, labels have a tendency to induce the behaviour that they describe, through the reinforcement of other people’s reactions (i.e., prognosis) [3]. Accordingly, “the person becomes the thing he is described as being” (Tannenbaum, as cited in [3, p. 3]). Those of us with severe-enduring courses of AN have often had complex diagnostic histories and been repeatedly given a poor prognosis for recovery prior to reaching the point of illness that may become severe enough to be considered ‘terminal’. These experiences significantly shape our sense of self, illness, sense of hope for recovery, and access/inaccess to and engagement in treatment. Receiving a terminal diagnosis *would* have emotional and psychological impact on a person and their loved ones, and create a new experience in shaping how an individual thinks and relates to their experience, the feelings and responses of others, choices and outcomes. To deny this aspect of experiencing a diagnosis is to limit an important area of discussion. Being told that there is no hope has been identified by individuals with SE-AN as a factor in maintaining the feeling that recovery is impossible, and part of the recovery phase of being unready or unable to change [7]. The experience of having a clinician instil hope and feeling understood was also identified as a key feature within the phase of a tipping point for recovery [7]. Hierarchies and stigma are known to exist among ED diagnoses and diagnostic subtypes, which may lead to shame, distress, and treatment avoidance [8–11], and these examples illustrate the impact of diagnoses and prognoses. Those of us with SE-AN may repeatedly experience severe medical crises and come close to death, and within these crises, we may experience world weariness, and desire for death. However, these feelings change at different points according to how we may conceptualise ourselves and our illness at different times. We may be attuned to a differing salience of our identities and narratives at different time points (e.g., a recovering identity, an advocate identity, an illness-salient identity), and have a different sense of healing (e.g. how harmful and accessible treatment is, and connection to hope).

Throughout my ED treatment in inpatient and outpatient services, I was repeatedly told by clinicians that I was ‘beyond clinical help’. I was told that my AN would be fatal, and that my death was predicted within weeks, months, or a few years. At times my awareness of continued clinical judgements of my poor SE-AN prognosis *itself* rather than my objective medical state and eating disorder behaviours has been the reason I have felt treatment is futile and reason I have not engaged in treatment. These messages and experiences had a significant impact on my beliefs about the possibility of recovery and my engagement with services. This included the following effects: (1) *Substantially decreasing* my sense of hope and belief in my ability to recover, (2) *Decreasing my attempts at help-seeking*, including seeking help in medical emergencies, (3) Increasing my sense of burdensomeness and shame (known risks for suicide) [12–16], (4) Decreasing my sense of meaningfulness in life (also a known risk for suicide, particularly in AN-R) [17–19], (5) Increasing my sense of isolation, (6) Triggering periods of not consuming food or water, (7) Rapidly facilitating a suicide attempt in hospital.

To further elucidate the final point, I was admitted to a general inpatient unit that specialised in ED treatment. The treating team decided I was to be treated for my AN only as the ‘primary threat to life’, and I was not to be treated for my co-occurring trauma, psychosis, depression or suicidality. I expressed to the team that I believed that there was nothing that could be done, that I believed I would never recover, and that I knew I was going to die. The team determined that I was a ‘unique case’ and kept me apart from the other ED patients, as my treatment was being approached differently, and it was recognised that other ED patients were voicing concern and distress about my condition. After weeks of nasogastric tube (NG) feeding, group therapy, and supervision, I experienced little to no improvement in my emotional wellbeing or weight. The treating team expressed that they felt I was ‘a hopeless case’ and that they believed there was ‘nothing more they could do’ to stop me from dying. At the time I felt there was some honesty and compassion in their agreeance with me that treatment was prolonging my suffering and that I would never recover. I felt both a deep sense of relief and profound grief. I was a multiple suicide survivor and had experienced the emotional damage in the wake of my previous suicide attempts. Now I felt I was being given a moral ‘permission’ or grace I needed to take my life—or at least, knew that my suicide would be met with understanding (as inevitable) and not with blame, anger, pain or guilt for anyone left behind. I could stop fighting—we all could. That was the relief—but what about the grief? In many ways, my AN has been a primal, embodied scream for safety “Please *god,* somebody help me”, and a way to meet that need when no help came. When treatments were inappropriate, invalidating or harmful, they reinforced the need for my AN, and reinforced the beliefs my AN was built on: that my life was worthless, that I was alone, that no help was coming. Having a treatment team in hospital agree with me that I was going to die of my illness and was beyond their help reduced my immediate suffering over 24 h; however it compounded my long-term suffering by confirming the deeply held beliefs my AN was built on, which stemmed from trauma. My supervision was reduced from 24/7 supervision to 10-min intervals; I attempted suicide, was found by staff members and revived through resuscitation and defibrillation, and was transported to another hospital where I remained in a coma for 2 weeks. After two months of rehabilitation and medical stabilisation, I was taken back to the ward where I had attempted suicide. The treating team informed me that I had traumatised multiple staff members, and would be given another two weeks of NG feeding and discharged. I was told that I was no longer welcome as a patient at their hospital, as treatment was futile and my intractability would be harmful to the recovery of others. Immediately after being discharged from hospital, I re-attempted suicide. This suicide attempt was thwarted by a brave stranger who intervened to save my life.

My concern is for the emotional impact of a ‘terminal’ AN diagnosis, particularly with respect to the underlying beliefs a person with AN may have, and whether or not this has been considered. Those of us with AN may have cognitive/temperamental schemas that have a significant role in how we communicate our feelings [20, 21], such as how we perceive and respond to a prognosis (i.e. 'terminal' AN). We may be highly sensitive to social experiences that relate to perceived abandonment/rejection, dependence/incompetence, and self-sacrifice/burdensomeness, and a 'terminal' diagnosis may trigger these maladaptive schemas and influence the way we engage with others regarding re-engaging with future treatment if our feelings change (i.e., threat-related responses such as surrender) [20, 21]. In response to fear, anxiety, sadness, hopelessness, anger and confusing feelings, we may frequently suppress our emotions and desire to ‘extinguish’ our bodies as the vessel of the threat [22]. While SE-AN may be partially driven by neurobiological factors that influence its tenacity (such as chronic neurobiological alterations in response to post-traumatic stress), psychological factors such as ingrained beliefs that may become reinforced throughout treatment (e.g., by prognosis) should also be assessed for their impact on the individual. Furthermore, I’m not convinced that a ‘terminal’ diagnosis is needed for access to hospice or palliate care where an SE-AN diagnosis and an individual’s specific case history may be sufficient.

## High-quality, multidisciplinary eating disorders care: equity, access and harm

Problems with accessibility, current treatment models, and negative treatment experiences must be considered when determining how someone with AN may progress to SE-AN. Early intervention in EDs is associated with better outcomes [23–28]. However, individuals with EDs face numerous barriers to accessing ED care and early intervention [29–32], and multiple issues influence treatment dropout. These include: (1) *Systemic Barriers* [33–41]; (2) *ED Treatment Barriers* [32, 33, 36, 39, 42–64]; and (3) *Personal Barriers* [34, 35, 41, 42, 46, 48, 51, 52].^1^ The proposed (3) ‘terminal’ AN criterion of “Prior persistent engagement in high-quality, multidisciplinary eating disorders care” therefore, may not represent the reality of ED treatment for a significant proportion of individuals who develop AN that progresses to SE-AN.

I experienced numerous traumatic experiences in inpatient and outpatient ED care, including: the use of physical, mechanical, and chemical restraints and confinement. I experienced sexual harassment by professionals during medical procedures such as ECGs, echocardiograms, medical exams. I also experienced discrimination, harassment, or assault while my bodily autonomy was compromised, such as while catatonic, chemically immobilised by antipsychotics and sedatives, and while on 24/7 eating disorder supervision. I experienced traumatic administration of medication: including being forcibly injected with medication while held down; having medication forced through my nasojejunal (NJ) tube while I was asleep (i.e., abuse of non-conscious state to force treatment; I had strongly stated I did not consent to the medication when awake); and being forcibly injected with sedatives and while immobilised, stripped and put in restraint clothing (anti-suicide smocks) and put in confinement. These experiences in inpatient and outpatient care compounded my trauma, and ultimately maintained my AN. Inpatient hospitalisations often led to deterioration in my mental health and increased my suicidality. The ED treatment I experienced had significant gaps with respect to important factors for my ED development and identity. The treatments I received were not trauma-informed [65, 66], and were not from strengths-based, affirmative approaches for gender and sexually diverse (LGBTIQ+) [64, 67], or neurodiverse people [61, 68–70]. These factors were significant, as traumatic experiences were etiological and maintaining factors in my AN development. Experiences of stigma, discrimination and identity-based violence had further contributed to embodied trauma and my use of disordered eating to cope. Clinicians’ conceptualisations of ED in treatment often directly erased and invalidated the meaning of my experiences,^2^ and these experiences reinforced my AN.

Problems with efficacy, quality, and access to existing treatments in AN highlight issues in defining what constitutes ‘good quality’ treatment, and therefore undermines any determination whether further treatment is ‘futile’. As existing treatments for SE-AN are poor and many people lack equitable access, Yager et al. [2] suggest that this should not preclude individuals with severe illness from access to end-of-life care and a compassionate, dignified death if so wished. This logic and argument becomes circular with regard to the proposed terminal diagnostic criteria—if one has not had access to ‘good quality’ treatment, how can treatment inequity exempt an individual from the terminal AN criterion: *Prior persistent engagement in high-quality, multidisciplinary eating disorders care* [1]?

The argument of ‘good quality care’ and inequity therefore, presents a dilemma: on the one hand, it may be recognised that current evidence-based treatments for SE-AN are insufficient, and greater equity is needed for access to ED treatment. When a small population of individuals with SE-AN become severely ill to a life-threatening degree, access to end-of-life care may alleviate profound suffering. However, it is important that this does not amount to lowering the threshold for accepting that inaccess to ‘good quality treatment’ facilitates the determination that an individual’s condition is ‘terminal’ and that they and their treating team accept that treatment is ‘futile’. This is particularly concerning for marginalised, underserved and disadvantaged populations who may face significant barriers and gaps in ED treatment. We must recognise the limitations of existing treatments and treatment access, seek to reduce suffering, and continue to protect vulnerable lives.

## Psychiatric euthanasia or physician assisted suicide (EAS): social justice

As argued by Yager et al. [2] of the proposed terminal AN diagnosis, there is a medical reality that a small population of individuals with severe SE-AN do not recover from their illness and die of medical complications. However, in facilitating access to EAS for individuals with SE-AN, while a ‘good death’ can be regarded as an issue of human rights and social justice, we must also consider the significant gender skew and psychiatric diagnoses within the use of EAS in some countries (i.e., eating disorders, personality disorders, trauma, autism) [71–73]. This gender and diagnostic skew is important as socio-cultural-political factors (i.e., gendered expectations, gender discrimination, sexual objectification, abelism) and traumatic experiences increase risk for ED development (e.g., [74–77]), and influence treatment dropout [78]. Hegemonic or marginalised gendered expressions and identity intersections (i.e., race, disability) may be met in psychiatric care with (dis)belief, afforded different agency, and given access to different options for therapeutic care [79, 80]. We must therefore interrogate what constitutes access to treatment, access to ‘quality’ care and access to recovery for individuals with EDs; particularly for those who experience oppression and marginalisation, and how this may influence progression from AN to SE-AN, treatment non-response and requests for EAS. Ultimately, these factors influence whose lives may be associated with better prognosis and who may be considered beyond help. While the clinical reality of severe SE-AN illness, resources and efficacy for ED treatment cannot be transformed overnight, this aspect of social justice has a place in the discourse of EAS, treatment futility and a ‘terminal’ diagnosis of AN. Clinicians who may seek to apply a diagnosis of ‘terminal’ AN to facilitate access to EAS should consider these dynamics to facilitate ethical decision-making.

## Treatment of co-occurring conditions

In response to Gaudiani et al.’s [1] case examples suggested to illustrate ‘terminal’ AN, Mack and Stanton [81] pointed out that the patients’ co-occurring conditions (i.e., depression, suicidality, obsessive compulsive disorder [OCD], anxiety) were not indicated as being appropriately treated, which may have profoundly impacted motivation, hope for recovery, and engagement in treatment. These complexities may also have shaped clinicians’ belief in treatment value or futility. As Mack and Stanton [81] also indicated, patients appeared to experience self-blame, and guilt for not recovering, possibly increasing their sense of hopelessness and desire for death. Self-blame and guilt for not recovering suggests an experience of ED stigma and self-stigma. Self-stigma is associated with increased ED severity, decreased treatment-seeking behaviour, decreased psychological wellbeing, low self-esteem, shame, and delayed recovery [82–85]. It was not specified in Gaudiani et al.’s case examples if the patients had received appropriate treatment for internalised ED stigma, shame, and feelings of guilt and burdensomeness that may have increased their sense of hopelessness for recovery and wish for death.

My schizoaffective diagnosis was similarly diagnosed as ‘intractable’ and ‘incurable’, after non-response to 8 anti-psychotic medications including clozapine and olanzapine; these messages of futility increased my sense of hopelessness, suicidality and led to treatment avoidance. Despite this prognosis, however, I have been in complete remission from psychosis over 5 years, without being on any of the anti-psychotic medications which previously led to severe side effects and loss of quality of life. This recovery was made possible after engagement with approaches that centred empowerment and autonomy including peer support work and work with a psychologist using recovery models and dialogical frameworks (i.e., Voice-Hearing approach, Chair Work, Schema Therapy; [86–91]), and trauma work. Following work on the untreated trauma and underlying beliefs that were driving my psychosis, I was able to engage with the same psychologist to work on my AN, and was able to reach a greater understanding of factors underlying my AN. Therapy was approached from a collaborative, trauma-informed, harm-reductive approach that affirmed my gender, sexuality and neurotype, and centred my strengths. Through this work, I have regained a sense of self-connection, meaning in life, and was able to continue university study, casual work, and to live independently. It was consistently predicted throughout my years of earlier treatment that I would be too debilitated by AN to engage in these life domains meaningfully. Using the harm-reduction approach, I have been able to maintain a BMI between 12–14 and maintain some medical stability during the COVID-19 pandemic (2020–2022), a period that also included management of personal crises (housing insecurity, becoming the target of stalking). As stated by Janse van Rensburg, who has lived experience of ED, harm-reduction, “… seeks to increase self-determination and promote social justice on an individual level. Rather than giving up completely, or forcing ED recovery, a harm-reductionist approach embraces the uncertainty of our times, and promotes a strengths-based dialectic perspective of EDs” [92]. A critical aspect of work with this psychologist has been my experience of having another person truly believe that my future includes a fully realised *recovered self*. While I still cannot imagine and do not believe in a full recovery, it has been a transformative experience to have another person believe in this future. By doing so, my psychologist’s belief has helped me to remain engaged in harm reduction despite my iatrogenic trauma and belief that I will die of my SE-AN. This relational dynamic is a pillar of my continued engagement: when I am weary of the struggle and longing for death, the knowledge of my psychologist’s belief acts almost as an antidote to AN. While AN can be experienced as perpetual delaying of life and self-preservation (“*I’ll eat tomorrow… I’ll go to the doctor next month… I’ll stop hurting myself when I stop being so worthless…*”) knowing another person holds belief for my recovery creates a perpetual delay of my intentions to disengage from treatment and engage in self-destructive behaviour (e.g., *“I can always stop treatment next session, but [my psychologist] believes I can be helped… I can still kill myself if this doesn’t work. But [my psychologist] believes in me, and I should be honest with them about how bad things are right now. Then if I die, I have honoured their commitment, and we can have peace knowing nothing more could have been done”*). This key therapeutic dynamic illustrates how having someone hold onto hope for recovery can lead to positive outcomes for complex co-occurring conditions in SE-AN (i.e., psychosis, self-harm) and help to maintain continued treatment engagement, particularly with respect to iatrogenic trauma.

## Alexithymia

Many of us with AN experience alexithymia [93, 94] and suppress our emotions as an emotional coping strategy [22, 95]. We may have difficulties identifying and expressing complex emotions to others particularly when overwhelmed by them, a difficulty that may be intensified by starvation effects and relational dynamics, including those of clinician-patient. This is critically important with respect to how our feelings of hope/hopelessness for recovery and continuing life itself may be felt and communicated. Alexithymia and differences in conveying emotion in AN may also be interlaced with atypical interoception [96–99] and trauma survival, as well as autism-trauma intersections [100–104]. For those of us who are also neurodiverse, it’s possible that alexithymia may also be interrelated with hyperlexia and pattern thinking, or a difficulty with conveying our emotions in the most accurate way when there are too many words available (information overload, leading to stress and disruption in communication) [105]. We may also experience a problem with double-empathy (mutual incomprehension) [106, 107], and become concerned about the other person over-empathising with our despair (hyper-empathy). Alexithymia is correlated with greater severity of disordered eating and suicidality in individuals with EDs [108, 109]. Alexithymia may increase suicidal ideation and risk through multiple interrelationships, including trauma, emotional dysregulation, social isolation, depression, atypical interoception [110–114], and engagement in self-injurious behaviour [115–117]. Further, expressive suppression in individuals with EDs is also linked to increased suicidal ideation [95].

When our emotions are overwhelmingly painful, AN offers a way to anaesthetise our pain and creates the illusion of a safe world to retreat into—this may include the pain and grief of being told we are beyond help. It may also mean we may struggle to put into words that we are feeling about death and what death represents to us. If we have histories of trauma and marginalisation, we may have experienced repeated silencing and invalidation, and find it difficult to express these complex feelings, or trust how they will be received. By living through SE-AN and its consequences, we may experience internalised shame, blame and self-stigma in association with our perpetual medical crises—which compounds the difficulty of expressing our pain and fear that we will not be met with compassion.

As part of my lived experience, longing for death or coming close to it often represented different things. In some of these experiences, suicidal drives or death wishes represented a need for rest and relief, and for my sense of danger to end. At other points, I wished for my entire existence to be erased, and my death drive was grounded in self-hatred and trauma. During others, I felt I was communicating my self-beliefs through my emaciation; my body was a vessel to demonstrate to others that I *knew* I was undeserving of breath, food, water, of occupying space in the world, and that I deserved to die. I felt that I was communicating somatically to the world *you don’t need to hurt and abuse me*—*I know I deserve to suffer, I will self-inflict it.* In other experiences, my death drive was related to retaining autonomy and control over my life and body and choosing the means of my suffering and death rather than feeling victimised, including trying to put measures in place to control what happened to my body after death. The individual meaning of AN, our underlying beliefs, and our death wishes are important, as they may also been unexplored within the ED treatment models that an individual has received. Alexithymia often created complexities in how I expressed (or did not express) changes in: my wish to engage/disengage from treatment, suicidality and illness perception. If a ‘terminal’ AN diagnosis such as that proposed by Gaudiani et al. [1, 2] is to be used, careful assessment of alexithymia and other communication patterns should be included in the assessment.

## Recovery

The definition of recovery^3^ from EDs itself lacks consensus, based in part due to conflicting criteria for relapse and recovery [118–120]. It has been suggested that research cannot be accurately conducted on a construct that is not clearly defined [121, 122]. Therefore, some authors have suggested that recovery definitions should be re-assessed based on lived experience recovery perspectives and motivations as opposed to clinical outcomes [121–123]. In contrast to the medical definition of recovery as an endpoint, lived experience recovery definitions centre a continuing process of personal transformation, reclamation of power, reconnection with the self and changing navigation of socio-cultural-political domains [124–128]. The socio-cultural and political environment for an individual is an important contextual factor in their healing and recovery (i.e., moralisation of food, desire, appetite and health; discrimination against diverse bodies, expressions, and neurodiversity [129–138]). The lack of diversity and inclusivity in ED recovery discourse and recovery archetypes must also be considered as part of how recovery from an ED is made possible and accessible [139]. Treatment approaches that centre recovery as an endpoint (rather than an ongoing or lifelong process) may lead to feelings of failure and hopelessness, and the perception of individuals with EDs and clinicians that treatment is futile. This must be considered with respect to how individuals with EDs may have engaged with ED treatment in the proposed ‘terminal’ AN criterion. Person-centred recovery and harm-reduction approaches may be particularly beneficial, especially for individuals with SE-AN [140–142], and recovery-model approaches for SE-AN may benefit both patients and clinicians [143].

Non-linear^4^ experiences of time within states of chronic illness and disability (known as ‘crip-time’, ‘queer time’ and/or ‘trauma time’[144–147]) may be useful to apply conceptually to the SE-AN context. Cripistemology understands experiences of space–time and place as being shaped by safety, survival practices and continuous crises [148, 149]. Through living with a chronic illness, those of us with SE-AN may experience time, space, health, our bodies, illness and recovery as non-linear, temporally ambiguous states. The sense of immediate danger posed by AN may be reduced through cyclical crises and living in a state of perpetual threat [149]. Idealized visions of recovery may therefore be less ‘real’ and relevant within these non-linear embodiments of space–time and ongoing survival threats. The phenomenology of how we experience space and time in SE-AN as a traumatic and chronic illness may partially explain the difficulty some of us experience in transcending the *need* we have for the illness as a survival practice in order to connect with our imminent need for treatment to protect our lives. This may be also be applicable to the complexity of capacity and impairment assessments, and our experiences of ambivalence. The non-linear embodiment of space–time within SE-AN is important to how we may also connect to diagnostic concepts and hope for recovery: at times healing and recovery feels more possible, even if only in a dreamlike, far-away concept within our non-linear time–space. This is where the narrative and diagnosis can be powerful, and where it becomes powerful for others to hold hope for us. I perceive SE-AN itself as a perpetual state of trauma, and both a suicidal and survival response. When I am experiencing my SE-AN as a survival response, it may appear to others as a suicidal, however wanting to survive and using AN to navigate the pain of my existence (even when aware of my risk to life) is different to being suicidal and using AN as a method of suicidal self-destruction. When recovery is made more ‘possible’ (e.g., through hope), as the inevitability of the threat of illness/death changes; I experience a corresponding change in my sense of time, future, and space. This influences my felt connection and sense of agency for my medical reality, and my motivation to address it.

However accessible recovery and healing is made and experienced, those of us with SE-AN may at many points be facing imminent death or severe medical complications that result in seeking out hospice or palliative care. Gaudiani et al. have argued that some with SE-AN may not recover, and may wish to end treatment, necessitating a terminal AN diagnosis [1, 2, 150]. When clinicians consider recovery and treatment, I would argue that the lived experiences of recovery as a concept and experience should be included in this discussion. This is not to argue completely against a terminal AN diagnosis, but to argue for examining an individual’s concept and experience of recovery and leaving treatment, including their embodied experience of space and time. It’s important that there is a mutual understanding of *how* an individual with SE-AN and clinicians define and understand recovery, ambivalence, and treatment futility, *why* they may occur and *what* their underlying meanings and relationships are, in order to apply a potential terminal AN diagnosis safely.

## Capacity to refuse treatment

The assessment of (in)capacity and use of coercion and involuntary treatment in AN is fraught with conflicting values and ethical questions of human rights [151–153]. It has been argued that AN creates a specific impairment in the domain of nutrition, and the creation of a ‘pathological value system’ that undermines autonomy and impairs capacity to engage in treatment, and that decisional capacity impairment is intrinsic in AN [154–156]. It has been suggested that incapacity in AN may be under-recognised, as capacity tests (i.e., the MacCAT-T) [157, 158] are unable to detect ‘pathological values’ associated with AN that may impair autonomy, such as valuing food avoidance and thinness [154, 159, 160].

In addition, it has been posited that an individual may be able to understand information regarding their condition (i.e., medical risk), but may be unable to use or meaningfully apply the information to themselves to inform their decision, and may demonstrate inconsistency between their stated goals and decisions [154, 156]. The outcome judgements of capacity assessments in AN treatment refusal, however, have been argued to contain logical errors that amount to a misapplication of functional capacity assessments [161, 162]. These include reliance on circular logic (i.e., presumption of incapacity based on the presence of AN itself) [163], and disregarding or not engaging with the subjective reasoning of the decision-maker with AN [161, 162]. The importance of distinguishing between incompetent and irrational decisions in treatment refusal has also been noted [164]. The presence of atypical motivations, values and beliefs is argued to be a flawed basis for determining incapacity [165]. Pozón states: “there is not enough information in the literature to determine when patients with EDs have sufficient skills to decide responsibly, although some studies indicate that, in general, such patients have considerable difficulties in deciding about their treatment” (165, p.22). Therefore, ethical decision-making models, position statements, and criteria to determine competence have been developed to address complex decisions in AN treatment [166–172]. It has been proposed that assessments of decision-making capacity in AN should include assessment of the specific decision and context, rigorous evaluation of cognitive factors, and the individual’s values, desires and identity, with due respect to the individual’s perspective, rights, and need for protection [165].

In some medical admissions, I would begin to experience the effects of nutritional restoration, and a differing perspective or renewed belief and desire for healing, as though I was ‘transcending’ the emotional need and embodied experience of AN. In some cases, this would occur before nasogastric feeding had been initiated. For example, after I had received 5 potassium IVs, I remember remarking to a nurse that I felt as though my brain and muscles had “come out of a trance, and come back online”. My mind and body felt stronger and more ‘alive’, and as I felt less medically fragile and close to death, and a spark of hope that my life could be saved. However, the ED treatment protocols were often so dehumanising and degrading, these effects were undermined, and my AN would be used as my primary coping strategy to increase my sense of safety and survival during and after treatment. In two involuntary admissions where I had received nutritional restoration via nasogastric and nasojejunal feeding, I was retrospectively grateful and recognised that my life had been in grave danger, aligning with reports in the literature [173]. The difference for me in these two admissions compared to others was that the approach had been compassionate and brief (prioritising collaboration, agency, dignity and freedom within the protocol, rather than coercion and punishment). Discharge also occurred after medical stabilisation, therefore re-feeding was less traumatic and medical care was experienced as less invasive, and fostered greater trust in the treating team. This allowed me to experience a greater sense of the overall good and need for the admission.

In many cases throughout my chronic treatment avoidance over the years, I longed for access to safe treatment. I enquired repeatedly to multiple hospitals regarding options for nasogastric or enteral feeding at home or as an outpatient without being subjected to the conditions of inpatient settings (i.e., dehumanisation, bathroom and shower supervision, punishment protocol, loss of agency, restraint and confinement, vulnerability to harassment and assault). I found the protocols and conditions within hospital ED programs incompatible with my safety. Therefore, I was persisting for improved quality of life whilst avoiding further inpatient hospitalisations. Eventually I stopped trying, and accepted that my SE-AN would not improve. However, despite the repeated messages of the ‘futility’ of my recovery from many clinicians and my own belief, I found a psychologist to work with me toward achieving greater value and meaning in my life. Although the *clinician* themselves was my choice of practitioner, at the time, I was mandated to seek treatment under a treatment order—I had given up. It took three years of building trust within the therapeutic relationship and trauma work to repair a lot of the despair and demoralisation I had experienced through earlier treatments enough to make any progress. After three and a half years, the treatment order was lifted, and I decided to continue with the treatment on a session-by-session basis. Because of it, I have lived for years longer than predicted, despite poor prognosis.

Is this an argument for the success of forced treatment, an example of impaired AN insight, or an example that prognosis can be wrong? It can be taken multiple ways. It illustrates the complexity of SE-AN and iatrogenic trauma, and the effort and resources it takes to continually work to repair. I am still severely ill with multiple medical complications, and still chronically avoid medical services. My healing is very gradual, and my medical complications may mean that I die before I reach a level of functional recovery by clinical definitions. The psychologist that has committed to working with me has been one among many professionals throughout years of services that gave me a different prognosis, and this was life-altering for my quality of life. I may not recover by the clinical curative definition, I may still die from my SE-AN, but I am *healing*. Some of us may not find the right set of circumstances to heal, or if we do so, we may find them late. Our autonomy to refuse harmful treatment must be safeguarded, but our wish and will to survive must be carefully assessed. Within the state of surviving chronic AN, we may be profoundly disconnected or desensitised to our ability to continue walking on a knife’s edge, and may feel that our suffering and the perceived unrecoverable severity of our condition is deserved (self-stigma), and therefore believe we must accept our circumstances.

As argued by Geppert [155], the concepts of treatment refusal and treatment-refraction is often conflated in the assessment of ‘futility’ in AN, and decisional capacity impairment in AN as a fundamental part of the illness may be inadequately considered [155]. This brings into question the nature of treatment refusal and ‘futility’ of care for SE-AN, as stated by Moreland: “That a severely ill patient would state that he or she wishes to live (and yet rejects the very treatment that is needed to achieve this goal) brings into question whether these patients have the capacity to refuse treatment” [174, p. 317]. Throughout many stages of my illness, refusing treatment has been important when inappropriate and traumatising treatment became harmful. It’s important to emphasise why I was refusing treatment and why continuing to live with AN became a ‘safer’ option to me: as previously stated, the treatment protocols and deliveries were invalidating to my identity and experiences and I experienced discrimination and trauma. However, being turned away from treatments due to the belief that treatment was ‘futile’ also created harm, hopelessness and avoidance of medical care, resulting in further medical complications and disability. The perception of futility within my treatment may have shaped and created a clinical prognosis and reality, and they also interacted with the schemas built on my trauma and maintaining my AN. In their application of a terminal AN diagnosis, Yager et al. [2] argue that patient autonomy is at the centre of their aim for *primum non nocere*. When ED treatments become traumatic and prolong our suffering, autonomy is critical to protecting our human rights and quality of life. However, it is important that clinicians do not conceptualise treatment refusal and treatment non-response as absolute states within severe and prolonged SE-AN. As Gaudiani et al. [1] have proposed a terminal AN diagnosis to have a fourth criterion for ‘*consistent*, *clear expression by an individual who possesses decision-making capacity that they understand further treatment to be futile*’, if an individual should change in their expression or wish for treatment, a diagnosis of terminal AN would no longer be justified. Just as diagnoses and prognoses cannot be absolutes, a terminal AN diagnosis (despite the implications associated with ‘terminal’) can be revoked. Care should be taken in clinicians’ determination of capacity for consent to AN treatment (including the use of capacity tests and ethical decision-making models), concept of treatment futility, and expression of their understanding that treatments will not be helpful for them. Clinicians should seek to understand what good or harmful care represent to the individual with SE-AN, and to ensure that they and the individual with SE-AN have a shared understanding of what recovery and treatment futility are. The clinician should also strive to understand expressions of hopelessness or wish to receive compassion, validation and hope that the individual may have, and how a diagnosis may fit into that need.

## Countertransference

Management of transference and countertransference are important features of how we experience ED care and how clinicians respond to us, particularly in enduring EDs, and must be effectively managed to reduce iatrogenic harms in treatment of SE-AN [175]. We may experience the receipt of compassion as painful, frightening, and untrustworthy [176, 177] which may be related to histories of trauma and ruptured attachment [178–180]. We may therefore find it difficult to develop trust in clinicians [181], reject and defend against receiving compassion [182–184], and reaffirm our belief and investment in AN as a safe harbour. In treating those of us with SE-AN, clinicians may be confronted with their own complex feelings regarding mortality, and a wish to ‘resolve’ our feelings of ambivalence and cognitive dissonance by prematurely ending treatment. I have experienced clinicians refusing or ending treatment as they felt that I was: “failure personified in the chair across from me” or that it had become “ghoulish” to witness my “living death”. A dietician refused to treat me until I “decided to commit to living”, and a multidisciplinary team informed me that my “refusal to recover” was “forcing us to participate in your suicide”. These experiences partially shaped my belief that I would not be able to recover by clinical definitions of full and complete recovery free from AN, and would likely die from AN or by suicide. I gave up on therapy, refused ambulance services and only experienced medical care through involuntary inpatient treatments after medical crisis, most of which I discharged myself from against medical advice.

Clinicians working with those of us who experience EDs such as AN may experience feelings of frustration, anger, hostility and fear, tiredness and a sense of being manipulated, hopelessness, helplessness and failure [181, 185]. These feelings may be intensely felt when we struggle to receive their compassion and help, do not respond to treatment, and our suffering becomes chronic [186]. For example, in a commentary on the sense of frustration and perceived futility in treating SE-AN, Groopman describes the conflicts of clinicians through a case illustration, including experiences of countertransference: “When I think about and imagine treating Angela—smart, skeletal, manipulative Angela, I am at my medical and moral wit’s end… I feel torn between violating her will by forcing her to eat (in the name of beneficence) and *getting rid of her* by discharging her (in the name of respecting her autonomy)” [187, p. 400] (emphasis added). He continues, “Angela is a deceiver… Should we trust anything she says… including that she wants us to leave her alone? ‘‘Palliation’’ for Angela might well consist of having others watch her slowly waste away, powerless to do anything. Perhaps that would be soothing to her. It would be torture to those forced to witness it. … We are not obligated to do whatever our patients ask of us. It is unconscionable to ask any caring person to watch this 18-year-old girl slowly waste away and expire from a disorder that has a 50 percent chance of full remission and at least an 85 percent chance of survival. In a case of this complexity, our standard ethical principles fail us” [187, pp. 402, 403].

Feelings of frustration, anger and helplessness toward AN patients may be mediated by clinicians’ years of experience working with ED patients and caseload, indicating possible effects of stress and burnout [185, 186]. Strong negative feelings of revulsion and disgust for ED behaviours may also be experienced [181, 186, 188]. Intense countertransferential feelings may be evoked when working with chronically ill, medically unstable AN patients who are at risk of death due to the impact of our EDs, which clinicians may perceive as an unwillingness to recover [181, 186, 188]. In these circumstances, it has been suggested that clinicians should be prepared to take compassionate and harm-reductive approaches (see [140, 174, 189]) that prioritise quality of life and reduce patients' isolation in suffering [181, 188]. As stated by Pies, invoking the term ‘futile’ may be reflective of negative countertransference indicating a clinician’s sense of frustration and helplessness when treating an AN patient who has not responded to treatment, rather than an objective medical determination [190]. Those of us with SE-AN may have been repeatedly turned away from services and been told that further treatment for us will be futile while we are still uncertain about whether or not further treatments may help or harm us; these experiences can be harmful and can shape a belief in the futility of treatment.

While a small population of severely ill SE-AN patients at an ‘end stage’ of illness may be argued to warrant a diagnosis of ‘terminal’ AN as suggested by Gaudiani et al. [1, 2], use of this proposed diagnosis should also bring careful consideration of dynamics of countertransference to ensure that it is medically warranted. Clinicians should also take the individual’s history and experiences of treatment into consideration, and explore internalised stigma, beliefs, expectation of abandonment, and sense of burdensomeness, guilt and shame in an individual’s sense that future treatments will fail.

## Ambivalence

Clinicians supporting SE-AN patients have described the importance and challenge of sustaining hope and the possibility of recovery for both patients and themselves, particularly individuals who have experienced multiple treatment models and/or have experienced treatment as harmful [143]. Those of us with SE-AN may lose and regain hope for recovery, and experience suicidality, world-weariness and wish for reprieve from our suffering through death at different points throughout the course of our illness, and ambivalence within these states of being. Euthanasia and physician-assisted suicide (EAS) is currently accessible for individuals with EDs in different countries [72, 191], however in the judgement of requests for EAS, an AN diagnosis has been sufficient to facilitate this process [72, 191]. In clinical and research contexts, ambivalence is overwhelmingly framed in a negative light, and may contribute to our feelings of shame, guilt and self-stigma. In ED treatment, it is rare for experiences of ambivalence to be understood as a self-protective and survival process, particularly for those of us with trauma histories, nor as representing an opportunity for reparation and restorative justice, healing and post-traumatic growth, to create deeper relational bonds and re-connection to life. If ambivalence is re-framed as a crucial process in renewing the drive toward life, the experience of ambivalence can be validated as a sign of hope, and encourage motivation and belief in recovery. As stated by Bergmans et al. [192, p. 139], ambivalence is “the dialectic holding of possibilities of living and dying at the same time… within the sphere of ambivalence is a kernel of hope that the person may not see or understand at the time”. They suggest that, “if one has spent a good part of life ‘living to die’, moving towards ‘dying to live’ is fraught with challenges of developing a new skill set and a different understanding of what living entails. This represents a first step in believing that things could be different—perhaps a new conceptualisation of one’s identity” [192, p. 139]. A ‘terminal AN’ diagnosis may extinguish hope and the conceptualisation of the identity of a healing self. We must consider the potential harm a ‘terminal’ AN diagnosis has in limiting transformative processes that may be represented by ambivalence, and that may occur through experiences of empowerment, validation, and increased sense of control. At the same time, the alternative exists that a ‘terminal AN’ diagnosis may facilitate the freedom of autonomy, liberation from suffering and a ‘good death’—both of these realities exist. It is possible to wish to be wholly free from AN itself, want to maintain the sense of protection it affords, want to be free from treatment models that are punitive and traumatic, and at the same time, wish to be rescued from physical and emotional danger.

Death and death wishes can be “understood relationally and attached to social and cultural meanings that change over time and across contexts” [193, p. 110]. Binge-eating, for example, may hold literalised embodied meanings of ‘being-in-the-world’ as felt states of release from existential states of emptiness and constraint into fullness and abundance [10]. AN may be experienced as a the desire to exist without needs and without a body, to contain the self while simultaneously self-annihilating and existing in a state between ‘being-and-not-being’ [194]. Suicidality and requests for EAS in AN may reflect an embodied ‘being-toward-death’, consisting of existential crises and body-self alienation [193]. This ‘being-toward-death’ includes detachment from the body and life; whereby AN is experienced as an external life of its own within the individual, and the body as a reflection of the life of the illness [193]. Externalisation of AN as a distinct entity or separate person rather than a clinical label has been used as a recovery tool to increase a sense of empowerment and mastery [195]. Some individuals experience AN as a voice, person or alternate self [196–198], and externalisation may be an important step in dialogical connection with the ‘healing/recovered’ self [7, 199]. Externalisation may be particularly helpful through transformative processes, including reclaiming identity [200] and reconstructing meaningful narratives such as fighting for life within AN, and reclaimed power [124]. For some, the ‘terminal AN’ diagnosis may erase these relational meanings of this narrative, and may construct the ‘healthy’ self that longs for survival as an ‘unrecovered identity’ that was unable to wrest power, identity, and meaning from AN as an external entity [124, 193]. The complex meanings of self-starvation and suicidality in AN, identity, ambivalence for recovery, and disengagement from treatment are important areas for consideration in the discourse of ‘terminal’ AN, and consideration of the best available approach to reduce suffering. For others, the ‘terminal AN’ diagnosis may provide validation and freedoms; the diagnosis and reality of this encapsulates the ambivalences, contradictions and dualism within AN itself.

In a qualitative study among healthcare professionals and volunteers involved in EAS in Belgium [201], some participants experienced EAS legislation and initiation processes as a double-edged sword. The option of EAS could have the effect of closing windows of opportunity for recovery and tipping their most vulnerable patients into experiencing a tunnel vision toward death, feeling discouraged and demoralised toward the likelihood of treatment success, applying for EAS, and being swept into a ‘death track’ [201]. In contrast, the option of EAS could lead to feelings of reassurance and encourage patients to refocus on treatment goals and to find relief from their suffering [201, 202]. This included patients reporting a decrease in self-destructive ideation and behaviour [202], and feelings of validation that their humanity and wish for death was recognised as more than solely psychopathology [202]. It’s important with respect to SE-AN and the proposed ‘terminal’ AN diagnosis to consider that these needs may occur for severely ill SE-AN patients who express suicidality, world-weariness and/or request for EAS.

In the Belgium study, for patients granted an EAS request, ambivalence was also experienced [202]. In one of the cases used by Gaudiani et al. [1, p. 6] to illustrate the proposed ‘terminal’ AN diagnosis, Jessica demonstrated repeated ambivalence about dying and the use of EAS, setting “multiple dates to use [medical aid in dying] over a couple of months and changed her mind as the date got closer”. The proposed ‘terminal’ diagnosis has been suggested to amount to a facilitation of the capability to complete suicide when an individual with SE-AN is experiencing ambivalence toward death [81]. Although the application of a ‘terminal AN’ diagnosis raises concerns, particularly regarding ambivalence, if the fourth criterion is applied appropriately and carefully, it should preclude individuals with SE-AN who experience ambivalence from accessing EAS. The first criticisms of the proposed criteria where that they were vague and could be misapplied to vulnerable individuals. Although Yager et al. [2] argue that the fourth criterion, if applied appropriately, would prevent this, the illustration of Jessica’s case gives some pause, as does the reception and criticism of the proposed diagnostic criteria. I would argue that ambivalence is inherent to the experience of AN, world-weariness and suicidality itself, and therefore care should be taken to ensure that individuals with SE-AN can have the space to express complex and conflicting feelings regarding life and death wishes, recovery, and treatment free of judgment and with the knowledge that multiple options remain should their wishes change. Clinicians and the public may benefit from further guidelines on the proposed diagnosis surrounding its intended application, particularly in assessing ambivalence and taking communication needs into account (alexithymia, trauma response patterns). Gaudiani et al. [1, 2] may also consider conducting a Delphi study on the proposed criteria, to gather data on the perspectives of individuals with lived experience of SE-AN, carers, and clinicians, including those working in acute, palliative, and hospice care settings.

I appreciate the reflections of Yager [150, 203] in advocating for weighing the reality of our suffering, our quality of life, and our humanity on a case-by-case basis when considering treatment continuation in AN. My experience has been that when a clinician took the approach of valuing my humanity, wishes, quality and meaning of my life above a clinical recovery and further inpatient treatments (taking a harm-reductive approach), it had the effect of increasing my quality and meaning of life. This is where I believe ambivalence must be addressed within this discourse; as a key mechanism of shift in liminality, hope, relationship to the self, others and the world, the self-and-ED relationship. At many points of the course of my life and enduring illness, I have felt that my life has become devoid of meaning, and that I had forced to endure suffering by repeated hospitalisations at the wishes of family members and my treating team. However, within this despair and life weariness; I also experienced ambivalence. Within my wish for an end to my suffering was the hope *for* hope. Having someone holding hope with and for me that my life is worth fighting for and that we can find a way to do so created the possibility for a *slow healing* that has brought me greater freedom and peace. My hesitancy for the proposed ‘terminal AN’ diagnosis is that it requires further refinement, consensus, and definition before it becomes widely applied.

## Conclusion

On reading the proposed criteria for ‘terminal’ AN [1], I felt a similar maelstrom of emotions that I felt after being repeatedly told I was beyond hope and help before I attempted suicide in hospital. On hearing that I was ‘beyond help’, I had initially felt a brief sense of relief, and a sense of receiving some form of recognition of the depth of my suffering. I knew that my death was expected by everyone, including myself, and this afforded me a release and freedom from the sense of guilt and burden of trying to fight for my life and recovery, a fight that I felt had become impossible. I felt a sense of peace and tranquillity: I could be free from the pain and distress of endless treatments and the desperation of the illness, and have control over the final moments of my life and death. These feelings were followed by a profound sense of grief and despair as the belief systems that my AN was built on felt as though they had been confirmed: my life was worthless, I was alone, and no help would ever come. Prior to my admission, I had stopped eating for six days and ceased my intake of water for three: I believed treatment was futile, and I wanted to die, preferably in a hospital, where it would be less traumatising for everyone. But even in the depth of this death wish, I retained an unspoken shred of hope that others could hold out hope *for me* that some*how* my life could still be saved. Hearing that nothing could be done to help me eliminated this hope, and heightened the suffering of having those beliefs confirmed. I didn’t wait to continue to starve to death, and acted immediately to take my life—being given that prognosis in that moment was a catalyst for suicide.

Last year and for five months of 2022, I would have met the proposed criteria for ‘terminal’ AN. These periods included the consistent belief that I would not recover and that treatment was futile, ceasing my intake of all food and nutritional supplements (which is my primary source of nutrition) for a week or more, and ceasing my intake of water for three days, accepting my own death, and asking my psychologist if they would be able to accept my death without feeling a sense of guilt, failure or responsibility. In response to these crises, my psychologist encouraged me to adopt curiosity within my pain and suicidality, to explore the meaning of healing and recovery in a harm-reductive framework, and to re-affirm their belief that I can recover, which restored a connection for me to the *possibility* of a healing/recovered future self. They continued to affirm my autonomy for ending treatment as we worked together in re-engaging in sessions after absences. If my psychologist had oriented themselves toward a diagnosis of terminal AN, my death could have occurred through starvation, suicide, and were I residing in a country with greater access to psychiatric EAS,^5^ perhaps through physician-assisted means [204–206]. Instead, through the foundation of trust and our therapeutic relationship, my life was preserved. Supporting my treatment re-engagement during these times was a notable achievement particularly in the context of my severe suicidality, my trauma complexity, co-occurring mental health concerns, and avoidance of medical treatment as a result of iatrogenic trauma.

In the judgement of requests for EAS, a ‘terminal’ AN diagnosis is not needed to facilitate the process of EAS in countries where it is currently possible. Individuals with mental health concerns have experienced the option of EAS legislation as increasing their motivation for recovery and decreased suicidality. EAS processes may also lead to experiences of ambivalence about death and a reduction in suffering through a sense of recognition and validation. Throughout my ambivalence for recovery, treatment engagement and dis-engagement, my light of hope vanished. What endured was my belief that my life was worth fighting for when others *held hope for recovery for me*. While I recognise the authors’ arguments for the benefits of their proposed diagnosis in facilitating access to compassionate end-of-life care, I believe that the potential for harm exists, and great caution must be used if and how this diagnosis is to be used. I argue that diagnoses and prognoses have important impacts on our feelings about our illness, futures, engagement with treatment, and the reactions and responses of others to us. Continued discourse around a potential terminal AN diagnosis is needed, and should include further refinement regarding the proposed criteria and its application, research with people with lived experience of SE-AN regarding the diagnosis, and development of guidelines for clinicians. The proposed diagnosis offers the opportunity for continued discussion about the lived experiences of SE-AN, ethical care and decision making, treatment response and engagement, illness staging, quality of life, end-of-life care, diagnosis and its impact. It is my hope that the discourse on severe SE-AN and terminal-stage illness approaches these topics with curiosity, and explores relational dynamics, schemas, alexithymia, ambivalence, and the transformative experience of having others believe that healing and a life of deep peace and meaning remains possible.

## Abbreviations

ACT: Acceptance and commitment therapy
AN: Anorexia nervosa
AN-R: Anorexia nervosa, restrictive subtype
BMI: Body mass index
CBT: Cognitive behavioural therapy
COVID-19: SARS-CoV-2 virus
CYMHS: Child and Youth mental health services
DBT: Dialectical behaviour therapy
EAS: Euthanasia and physician-assisted suicide
ED: Eating disorder
EDs: Eating disorders
ICU: Intensive care unit
IPT: Interpersonal therapy
LGBTIQ+: Diverse affective and sexual orientation and gender identity, including identification as lesbian, gay, bisexual, transgender, non-binary, gender diverse, intersex, queer, questioning, agender, aromantic, asexual, Two-Spirit, pansexual, plurisexual and further terms of identification that refer to non-heterosexual and non-cisgender sexual/affective and gender identities and experiences
MAID: Medical assistance in dying
MCT: Metacognitive therapy
NG feeding: Nasogastric feeding
OCD: Obsessive compulsive disorder
SE-AN: Severe-enduring anorexia nervosa

## Footnotes

1 (1) Systemic Barriers: problems with service availability/accessibility; inequity; and long waiting lists; consistency and continuity problems; financial and insurance barriers; BMI and weight-based cut-offs and triage. (2) ED Treatment Barriers: problems with quality of care and care delivery, insufficient expertise in ED treatment; lack of holistic approach, lack individualised care and lack of collaborative involvement in treatment; over-focus on weight and eating to the neglect of psychological/emotional concerns; invalidation/trivialisation; dehumanisation and degradation; rigid procedures and punishing and coercive approaches; treatment approaches that are not culturally appropriate or inclusive of diversity. (3) Personal Barriers: Minimal social support and family tension and pressure; illness denial and cognitive dissonance; lower motivation [fear of losing control and need to maintain self-sovereignty; valuing aspects of the ED]; and internalised stigma and shame.

2 For example, my autistic communication patterns and movement (low eye contact, stimming) were often misinterpreted through neurotypical and ableist lenses, or interpreted as being due to AN, such as: ‘regression’, being ‘unwilling to grow up’, ‘undermining staff’, and ‘fidgeting to trying to covertly lose weight’. I was often forced to make eye contact through threats, coercion, or physical force, with led to sensory overload, autistic burnout, shutdown, and more severe disordered eating. I was threatened with extended force-feeding or bedrest if I used stimming behaviours, despite this being an adaptive behaviour for autistic people to self-soothe and self-regulate; this also led to a deterioration in my AN, and a greater sense of threat and distrust in medical and mental health services. Similarly, I experienced repeated invalidation and discrimination with respect to my identity as a gender and sexually diverse person. My disordered eating experiences in relation to gender dysphoria and my need for gender affirmation and gender euphoria were positioned as being ‘wanting to be helpless and remain childlike’, ‘being afraid of your identity’, ‘fear of relationships and sexuality’, ‘fear of pregnancy and childbirth’, ‘probably trauma-based’, ‘the anorexia making excuses’ and ‘being confused’. My attempts to refute this were positioned as being ‘treatment resistant’, ‘unwilling to recover’ and ‘undermining the treatment protocol’.

3 Two predominant models of recovery are broadly examined within the research literature. One of these models, the biomedical model, defines recovery by the absence of ED symptoms, and a return to former functioning (objective ‘cure’ of AN) [118]. The other model, the recovery model, understood by those with lived experience, defines recovery as an ongoing process that involves personal transformation, empowerment, and the improvement of quality of life, where disordered eating and functional impacts may still be experienced within a meaningful life [119]. A recovery definition that emphasises the importance of physical, behavioural, and psychological features has also been defined [120]. The recovery model has been applied to AN, however the biomedical model is the dominant recovery definition and approach used within the clinical treatment and research of EDs.

4 Crip-time as a non-linear experience within chronic illness and disability may be used to give context to SE-AN embodiment of illness/recovery and discourse around ‘intractability’ and ‘futility’. As described by Samuels; “Some of us contend with the impairments of old age while still young; some of us are treated like children no matter how old we get. The medical language of illness tries to reimpose the linear, speaking in terms of the chronic, the progressive, and the terminal, of relapses and stages. But we who occupy the bodies of crip time know that we are never linear” [147].

5 The Northern Territory, Australia, was the first jurisdiction in the world to legalise voluntary euthanasia (the Northern Territory Rights of the Terminally Ill Act 1995 (NT), May 25, 1995), however that law was overturned soon after by the by the Commonwealth Government’s Euthanasia Laws Act 1997 (Cth). The Australian state of Victoria is the only state in Australia where voluntary euthanasia is legal [205]. It is not legal if psychiatric illness or disability are the sole grounds for the request, however having a psychiatric illness or disability does not deem an individual ineligible from euthanasia if all eligibility criteria are met [205]. The Victorian protocol for voluntary euthanasia has a narrow scope of eligibility and 68 safeguards, and has been described as “the safest most conservative scheme in the world” [206].
